# Strategic Management of Design and Conceptualization Factors for Wearable Postural Rehabilitation Devices: A Causal Interdependency Analysis

**DOI:** 10.3390/biomimetics11060386

**Published:** 2026-06-01

**Authors:** Anghel Constantin, Cristian Radu Badea, Roxana-Mariana Nechita, Corina-Ionela Dumitrescu, Bogdan Marian Verdete, Florentina Badea, Sorin Ionuț Badea

**Affiliations:** 1 Department of Mechatronics and Smart Measurement, National Institute of Research and Development in Mechatronics and Measurement Technique, 021631 Bucharest, Romania; anghel.constantin@incdmtm.ro (A.C.); sorin.badea@incdmtm.ro (S.I.B.); 2 Department of Entrepreneurship and Management, Faculty of Entrepreneurship, Business Engineering and Management, National University of Science and Technology POLITEHNICA Bucharest, 060042 Bucharest, Romania; roxana.nechita@upb.ro; 3 Doctoral School of Entrepreneurship, Engineering, and Business Management, Faculty of Entrepreneurship, Business Engineering and Management, National University of Science and Technology POLITEHNICA Bucharest, 020943 Bucharest, Romania; 4 Department of Biomedical Mechatronics and Robotics, National Institute of Research and Development in Mechatronics and Measurement Technique, 021631 Bucharest, Romania; 5 Department of Economics, Faculty of Entrepreneurship Business Engineering and Management, National University of Science and Technology POLITEHNICA Bucharest, 060042 Bucharest, Romania; corina.dumitrescu@upb.ro; 6 Department of Machine and Production Systems, Faculty of Industrial Engineering and Robotics, National University of Science and Technology POLITEHNICA Bucharest, 060042 Bucharest, Romania; bogdan.verdete@upb.ro; 7 Robodictive SRL, 110253 Pitești, Romania; 8 Department of Strategic Marketing, National Institute of Research and Development in Mechatronics and Measurement Technique, 021631 Bucharest, Romania; florentina.badea@incdmtm.ro

**Keywords:** wearable sensors, postural rehabilitation, DEMATEL, rehabilitation

## Abstract

The implementation of wearable systems in postural rehabilitation offers new perspectives for continuous monitoring, yet their success depends on the interaction between technical parameters and clinical requirements. This study analyzes clinical performance factors to identify strategic levers determining recovery effectiveness. It examines indicators such as postural deviation detection, sensitivity to minor motion changes, suitability for home monitoring, continuous monitoring capability, clinical relevance of extracted parameters, and the capability to assess patient progress over time. Using the DEMATEL methodology, the study highlights influences among these factors in a clinical context. This structural analysis separates primary drivers from rehabilitation outcomes. To refine the analysis, the MICMAC method classifies factors by driving and dependence power, distinguishing determinant, relay, dependent, and autonomous variables. The approach provides an objective basis for managers and designers to prioritize resources toward functionalities with the greatest systemic impact on patient progress. The combined DEMATEL–MICMAC framework enhances decision-making by linking causal relationships with clear hierarchical categorization. The findings may support the integration of wearable technologies into rehabilitation practice by identifying the clinical performance factors with the strongest influence within the system.

## 1. Introduction

Healthcare systems are undergoing a gradual shift toward remote monitoring, personalized rehabilitation, and data-driven clinical decision-making [1]. This shift is supported by rapid advances in wearable sensor technologies [2], which enable continuous measurement of human motion in real-world environments [3]. In recent years, inertial measurement units, flexible sensors, and compact wireless platforms have made it possible to track posture, joint angles, and activity patterns outside specialized laboratories [4,5,6]. These technological developments have opened new opportunities for improving the assessment and management of musculoskeletal disorders, neurological conditions, and post-injury rehabilitation [7].

Postural disorders and movement impairments represent a widespread clinical concern across different age groups. Prolonged sedentary behavior, increased screen time, and aging populations contribute to a higher prevalence of poor posture, spinal misalignment, and functional movement limitations [8,9]. Such conditions are associated with chronic pain, reduced mobility, and decreased quality of life [10]. Early detection and continuous monitoring of postural deviations can support preventive care and enable more effective rehabilitation strategies. However, in standard clinical practice, posture assessment is often performed during short clinical visits using visual inspection, goniometry, or laboratory-based motion capture systems [11]. These approaches provide only limited snapshots of a patient’s functional behavior and may fail to capture variations that occur during daily activities [12].

Wearable sensor systems have been proposed as a practical solution to this limitation [13]. By allowing data collection during everyday activities, these devices can provide clinicians with a more realistic picture of patient movement patterns and postural habits [14]. Several studies [15,16,17] have demonstrated that wearable inertial sensors can estimate joint angles, detect abnormal postures, and provide real-time feedback to users. Despite these advances, the adoption of wearable systems in routine rehabilitation practice remains uneven. Many prototypes show promising technical performance in controlled environments, yet their clinical use is still restricted to pilot studies or experimental deployments [18].

This situation reveals a complex problem that emerges at the intersection of engineering, clinical practice, and patient behavior. The performance of wearable systems in rehabilitation does not depend on a single characteristic [19,20,21,22,23]. Instead, it is shaped by multiple interrelated factors such as the ability to detect postural deviations, the sensitivity of the sensors to subtle movements, the usefulness of the system for home monitoring, and the degree to which the measurements [24,25,26] align with standardized clinical methods. Additional aspects, including real-time feedback, continuous monitoring, and the clinical relevance of extracted parameters, also influence the overall value of such systems. Because these factors interact with each other, improvements in one area can amplify or limit the benefits of another [27].

The problem is most visible in ambulatory rehabilitation and home-based therapy programs [28]. In these settings, clinicians have limited direct supervision over patients, and adherence to prescribed exercises is often inconsistent [29]. Without objective monitoring tools, therapists rely on patient self reports or periodic clinical assessments, which may not reflect actual behavior. As a result, incorrect posture or compensatory movements can persist over long periods, reducing the effectiveness of rehabilitation and increasing the risk of secondary complications [30]. Patients, therapists, and healthcare providers are all affected by this limitation. Patients may experience slower recovery, therapists may lack reliable data to adjust therapy plans, and healthcare systems may face higher long-term treatment costs [31].

Although a growing body of research investigates wearable sensors for posture monitoring, several gaps remain in the existing literature [1,18,31]. Many studies focus on the development and validation of individual devices or algorithms, with emphasis on accuracy metrics or hardware design [32,33]. Other works evaluate user acceptance or usability aspects in isolation. Fewer studies [18,34] address the relationships between the different clinical performance factors that determine whether a wearable system becomes a useful rehabilitation tool in practice. As a consequence, there is limited understanding of which factors act as primary drivers of system effectiveness and which factors are mainly influenced by others. Existing studies on wearable rehabilitation systems also focus predominantly on device validation or sensor performance, while limited attention has been given to the structural relationships between clinical and functional performance factors through DEMATEL-MICMAC approaches.

Traditional evaluation methods used in this field often rely on simple performance comparisons or ranking techniques [35]. These approaches can identify which system performs better under certain conditions, yet they provide limited insight into the causal relationships between influencing factors. In complex healthcare technologies, where technical characteristics, clinical requirements, and user interaction are closely linked, an analytical approach that captures interdependencies between factors is required. Multi-attribute decision-making methods offer a structured way to analyze such situations [36,37], but not all methods are suited to reveal causal influence patterns or to clearly classify factors based on their systemic role.

In this context, the Decision Making Trial and Evaluation Laboratory (DEMATEL) method offers a suitable analytical framework. DEMATEL was developed to model cause–effect relationships in complex systems where multiple factors influence each other [37]. The method transforms expert evaluations into a structured matrix that highlights both the strength and direction of influence between variables. Through the computation of prominence and relation indices, DEMATEL distinguishes between factors that primarily drive the system and those that are mainly affected by others. This capability is particularly relevant for the evaluation of wearable rehabilitation technologies, where design features, clinical performance, and user interaction form a tightly connected network of influences [38]. After applying the DEMATEL method, the study also uses the Matrice d’Impacts Croisés Multiplication Appliquée à un Classement (MICMAC) method. This step classifies the factors according to their driving power and dependence and shows which factors serve as main levers for change and which ones depend more on the rest of the system. This complementary step extends the analysis by classifying the factors into driver, relay, dependent, and autonomous groups based on their driving and dependence power, thereby providing a clearer hierarchical interpretation of their roles within the system.

The main objective of this study is to analyze the structural relationships between key clinical performance factors that determine the effectiveness of wearable sensor systems for posture monitoring in rehabilitation. The study focuses on eight factors that describe the clinical and functional value of such systems, including postural deviation detection capability, sensitivity to minor motion changes, suitability for home monitoring, compliance with standardized measurement methods, real-time feedback capability, continuous monitoring capability, clinical relevance of extracted parameters, and the ability to assess patient progress over time. The factors were identified through a review of recent studies on wearable sensing systems, rehabilitation monitoring, and clinical motion assessment. After the initial selection stage, overlapping concepts were removed, and the remaining factors were refined through expert discussion in order to ensure conceptual clarity, clinical relevance, and independence between variables.

The research is guided by the following question: what are the causal relationships between the main clinical performance factors of wearable posture monitoring systems, which of these factors act as primary drivers that shape the overall effectiveness of such technologies in rehabilitation contexts and how can these factors be structurally classified according to their level of influence and dependence? By addressing this question, the study aims to provide a clearer understanding of how different system characteristics interact and how improvements in specific areas may lead to broader clinical benefits.

To answer this question, the study applies the DEMATEL method using evaluations provided by a panel of experts with experience in biomedical engineering, rehabilitation, and wearable system design. The method is used to construct the direct relation matrix, normalize the influence values, and derive the total relation matrix that captures both direct and indirect effects. The resulting cause–effect diagram allows the identification of the most influential factors and the visualization of their interconnections. Subsequently, the MICMAC method is applied to the same total relation matrix in order to classify the factors based on their driving and dependence power, ensuring a coherent and integrated analytical framework without requiring additional expert input.

The expected outcome of this research is a prioritized map of clinical performance factors that highlights the key levers for improving wearable posture monitoring systems. Such information can support designers in focusing development efforts on the features that have the strongest systemic impact. It can also assist clinicians and decision makers in understanding which system capabilities are most critical for achieving reliable and meaningful rehabilitation monitoring. By combining DEMATEL and MICMAC, the study not only identifies causal relationships but also provides a structured classification of factors, enhancing the practical relevance of the results for decision-making and system design. Since the analysis is based on expert evaluation and structural modeling, the findings should be interpreted as decision support results intended to guide future system development and clinical investigation.

## 2. Theoretical Framework

Wearable sensor technologies have gained significant attention in recent years as tools for continuous monitoring of human movement and posture. Their development is closely linked to advances in microelectronics, wireless communication, and signal processing [39]. These technologies allow the collection of biomechanical data outside controlled laboratory environments, which is particularly valuable in rehabilitation, where patient behavior during daily activities plays a major role in recovery outcomes [1]. As a result, wearable systems are increasingly investigated as alternatives or complements to traditional motion capture systems used in clinical assessment.

The use of wearable sensors in posture monitoring involves the measurement of body segment orientation, joint angles, and movement patterns through inertial measurement units, pressure sensors, or flexible sensing elements [4]. These systems aim to provide objective and quantitative data that can support clinicians in diagnosing postural disorders, tracking rehabilitation progress, and evaluating the effectiveness of therapeutic interventions. Despite the growing number of prototypes and pilot studies, the translation of these systems into routine clinical practice remains limited [40]. This situation indicates the presence of multiple factors that influence their real-world usefulness and acceptance.

To understand these factors, it is necessary to examine the wearable monitoring system as a complex socio-economic technical solution rather than a simple electronic device [41]. The effectiveness of such systems depends on the interaction between sensor performance, data interpretation, clinical relevance, user behavior, and economic feasibility, including implementation costs, maintenance requirements, and accessibility for healthcare providers and patients. A system that performs well from a technical perspective may still fail to provide meaningful support in rehabilitation if the collected data are not clinically interpretable or if the monitoring process is not suitable for use in home environments. Therefore, the evaluation of wearable posture monitoring systems requires a framework that takes into account several interdependent clinical performance factors.

Based on a structured review of recent literature in wearable sensing, biomechanics, and rehabilitation engineering, eight key factors were identified as essential for describing the clinical performance of wearable systems used for posture monitoring. The initial selection included a broader group of technical and clinical indicators reported in previous studies. Factors with overlapping meanings or limited relevance for rehabilitation monitoring were excluded during the screening stage. The remaining factors were refined through expert consultation in order to improve conceptual clarity and reduce redundancy between variables. These factors reflect the ability of the system to detect relevant postural deviations, capture subtle changes in movement, support remote monitoring, and provide data that are consistent with established clinical measurement methods. Table 1 presents the factors included in the analysis.

The identification of the eight factors was based on an analysis of the specialized literature. The authors examined several scientific articles to extract an initial pool of relevant indicators. These factors were then reviewed and validated by a group of researchers specializing in assistive technologies, with a focus on wearable sensors. This validation process ensured that the final selection included the most significant variables for the study, filtering out redundant elements. The final list of eight factors resulted from the consensus between the evidence found in the literature and the practical expertise of these researchers in the field of wearable technology.

The first factor, postural deviation detection capability (F_1_), refers to the ability of the wearable system to identify abnormal or non-neutral body positions that may be associated with musculoskeletal strain or functional impairment [1,10]. In rehabilitation contexts, this capability is essential for recognizing incorrect posture during daily activities and for alerting both patients and clinicians when deviations exceed acceptable thresholds. Accurate detection supports early intervention and helps prevent the reinforcement of harmful movement patterns [1,10,18].

Sensitivity to minor motion changes (F_2_) describes how well the system can detect small variations in body orientation and movement amplitude. Rehabilitation often involves gradual improvements that occur over extended periods. If a wearable device lacks sufficient sensitivity, these small but clinically important changes may remain unnoticed [3,7]. This can lead to an incomplete or misleading assessment of patient progress. High sensitivity allows clinicians to observe subtle improvements or deteriorations and to adjust therapy plans accordingly [3,4].

Suitability for home monitoring (F_3_) reflects the extent to which the wearable system can be effectively used outside clinical facilities [8,9]. Many rehabilitation programs rely on exercises performed at home, where direct supervision by therapists is not available. A system that is difficult to use requires frequent recalibration or depends on complex setup procedures may discourage patients from using it regularly. In contrast, a system that is easy to operate and comfortable to wear is more likely to support long-term adherence and to provide continuous streams of data from real-life conditions [30].

Compliance with standardized measurement methods (F_4_) refers to the degree to which the data generated by the wearable system align with those obtained through established clinical tools such as optical motion capture systems, goniometers, or validated clinical scales [4,6]. This factor is important because clinicians rely on standardized methods to ensure that measurements are reliable and comparable across patients and sessions [12]. If wearable sensors produce results that diverge significantly from these reference methods, their clinical credibility may be questioned.

Real-time feedback capability (F_5_) represents the system’s ability to provide immediate information to the user regarding posture or movement quality [5,26,27]. In rehabilitation, timely feedback can support motor learning and encourage patients to correct their posture during task execution. This feature transforms the wearable device from a passive monitoring tool into an active training aid. Visual, auditory, or haptic feedback mechanisms can be used to inform users when their posture deviates from predefined limits [42].

Continuous monitoring capability (F_6_) describes the system’s capacity to collect data over extended periods without interruption [5,15]. This factor is related to aspects such as battery autonomy, data storage, and wireless transmission stability. Continuous monitoring allows clinicians to observe variations in posture throughout the day, including during work, leisure, and rest periods [5,8]. Such information can reveal patterns that are not visible during short clinical assessments.

The clinical relevance of extracted parameters (F_7_) refers to the extent to which the metrics computed from raw sensor data correspond to variables that are meaningful for therapeutic decision-making. Wearable sensors generate large volumes of data, including acceleration, angular velocity, and orientation estimates [2]. These raw signals must be transformed into interpretable indicators such as trunk inclination angle, symmetry indices, or movement variability measures [13,19]. If the extracted parameters do not correspond to clinically recognized indicators, their practical value remains limited.

The final factor, capability to assess patient progress over time (F_8_), addresses the system’s usefulness in longitudinal evaluation. Rehabilitation is a process that unfolds over weeks or months. Clinicians require tools that can track trends, highlight improvements, and identify plateaus or regressions [8,15]. A wearable system that supports the storage, comparison, and visualization of historical data can assist in documenting treatment outcomes and in communicating progress to patients [15,28].

These eight factors represent different but interconnected aspects of the clinical performance of wearable posture monitoring systems. Improvements in one factor can influence the effectiveness of others. For example, higher sensitivity to minor motion changes can enhance the detection of postural deviations, while continuous monitoring capability can strengthen the assessment of patient progress over time. Because of these interactions, it is not sufficient to evaluate each factor in isolation.

The theoretical framework adopted in this study treats the wearable posture monitoring system as a network of interdependent clinical performance factors. This perspective supports the use of analytical methods that can capture causal relationships and influence pathways between variables. By structuring the problem in this way, the study aims to move beyond simple performance comparisons and to develop a deeper understanding of how different system characteristics contribute to the overall effectiveness of wearable technologies in rehabilitation contexts. The framework also supports the integration of DEMATEL and MICMAC methods by linking causal influence analysis with the classification of factors according to their driving and dependence power.

## 3. Materials and Methods

The present study investigates the relationships between clinical performance factors of wearable sensor systems used for posture monitoring in rehabilitation. The problem addressed in this research involves multiple criteria that influence each other and jointly determine the usefulness of these technologies in real clinical settings. Because of this complexity, a multi-criteria decision-making (MCDM) approach was selected as the analytical framework of the study.

MCDM methods are commonly applied in engineering and healthcare research when a system is influenced by several factors that cannot be evaluated independently [37]. In the context of wearable rehabilitation devices, aspects such as measurement reliability, feedback capability, and suitability for home use interact with each other and may produce indirect effects. Traditional evaluation methods based on isolated performance indicators are not able to capture these interdependencies. For this reason, the study required a method capable of analyzing both the importance of factors and the relationships between them.

Among the available MCDM techniques, several methods such as the Analytic Hierarchy Process (AHP), Analytic Network Process (ANP), and Best Worst Method (BWM) are frequently used for ranking or weighting criteria. These approaches are effective when the objective is to establish a hierarchy of importance [37,44]. However, they provide limited information about the direction and strength of influence between criteria. Since the aim of this study is to understand how clinical performance factors of wearable systems influence each other, a method that explicitly models causal relationships was required.

For this purpose, the DEMATEL method was selected. DEMATEL is designed to represent complex systems as networks of cause-and-effect relationships. It allows the identification of factors that act as drivers and those that are mainly influenced by others. This characteristic makes DEMATEL suitable for analyzing the structure of wearable rehabilitation technologies, where technical, clinical, and user-related aspects form a connected system rather than independent variables [38].

The DEMATEL method relies on expert judgment to evaluate the degree of influence between factors. The quality of the results therefore depends on the experience and relevance of the selected experts. In this study, the expert panel was composed of specialists involved in the development of wearable monitoring equipment, including activities related to system design, hardware and software integration, and technical documentation. Particular attention was given to selecting experts whose work is directly connected to patient-centered design, since wearable rehabilitation systems must be adapted to clinical use and patient interaction.

A minimum professional experience of ten years in the field of wearable or biomedical device development was established as a selection criterion. This threshold was chosen to ensure that participants had sufficient exposure to the complete development cycle of such systems, including requirement analysis, design iterations, validation procedures, and risk assessment. In engineering practice, a period of ten years is often considered adequate for professionals to encounter both frequent and low-probability issues, such as rare failure modes, usability challenges, or regulatory constraints. Experts with shorter experience may have only partial exposure to these aspects, which could limit the reliability of their judgments regarding system-level interactions.

A total of 11 experts who met these criteria agreed to participate in the study. This number is consistent with common practice in DEMATEL applications [38,45], where a small group of knowledgeable specialists is preferred over a larger group with less specific expertise. All participating experts had professional experience equal to or greater than ten years in wearable technology development, biomedical engineering, rehabilitation technology, or related applied engineering activities.

Data collection took place in March 2026 using a methodological questionnaire that represented the unaggregated matrix of direct relationships. The matrix contained the eight clinical performance factors identified in the theoretical framework. Each expert was asked to assess the degree to which one factor influences another. Since eight factors were considered, each expert completed 64 evaluations, corresponding to all possible ordered pairs of factors.

The influence between factors was rated using a five-level scale:

- 0—no influence;
- 1—low influence;
- 2—moderate influence;
- 3—strong influence;
- 4—very strong influence.

The individual matrices provided by the experts were aggregated by calculating the arithmetic mean for each element. In addition to the arithmetic mean values, the standard deviation was calculated for each element of the direct relation matrix in order to evaluate the dispersion of expert judgments and the level of agreement among participants. The standard deviation values were computed based on the evaluations provided by the 11 experts for every pairwise relationship between the analyzed factors. Lower standard deviation values indicate stronger convergence of expert opinions, while higher values reflect greater variability in the perceived influence between factors. The resulting standard deviation matrix is presented in Appendix B.

The present study involved a group of specialists with extensive experience in developing monitoring systems and wearable sensors. These experts possess the necessary knowledge to anticipate the functionality of a product starting from the design phase. Since this research aims to provide a practical tool for designers of such equipment, the selection of participants focused on individuals with real-world expertise in the field. This approach ensures that the findings are transformed into an open-access instrument that is useful for the industry.

The economic and clinical perspectives were integrated into the study as they form an inherent part of the experts’ professional vision. Beyond completing the questionnaires, the specialists shared their insights during individual training sessions organized for this research. Their long-term involvement in various clinical trials allows them to understand patient needs and satisfaction levels throughout the entire life cycle of a product. Furthermore, the research team included an economist who collaborated with the experts to ensure that the financial and sustainability aspects were properly addressed and reflected in the final model.

The resulting matrix represents the collective opinion of the expert panel and is denoted as the initial direct relation matrix A (Appendix A). The direct relation matrix A is expressed as:

(1) $$A=\left\lfloor a_{ij}\right\rfloor$$

where i and j take values from 1 to n, and n represents the number of factors included in the study. In this research, n equals 8. Each element $a_{ij}$ represents the average score assigned by the experts to describe how strongly factor i influences factor j. The diagonal elements $a_{ij}$ are equal to zero because a factor is not considered to influence itself. This matrix constitutes the starting point of the DEMATEL calculations and contains only direct influences reported by the experts.

The values in matrix A may exceed the range required for stable matrix operations. To avoid numerical instability and to ensure that the influence values are comparable, the matrix must be normalized. This is achieved by multiplying A with a scaling coefficient k, resulting in the normalized matrix Y:

(2) Y=A·k

The coefficient k is calculated using the maximum row sum of matrix A:

(3) $$k=\frac{1}{{max}_{1\leq i\leq n}\left(\sum_{j=1}^{n}a_{ij}\right)}\;(i,j=1,\;2,\;\ldots \;,\;n)$$

This operation ensures that the largest total influence exerted by any factor becomes equal to 1 after normalization. As a result, all elements of matrix Y are constrained to the interval between 0 and 1. This step is necessary because the next phase of the DEMATEL method involves matrix inversion, which requires the matrix values to remain within a stable numerical range.

Matrix Y represents only the direct influences between factors. In complex systems, however, a factor may influence another indirectly through one or more intermediate factors. To capture these indirect paths, DEMATEL computes the total relation matrix T, which combines both direct and indirect effects. The total relation matrix T is calculated using the formula:

(4) $$T=Y\cdot {\left(I-Y\right)}^{-1}$$

In this expression, I is the identity matrix of the same dimension as Y. The term $\left(I-Y\right)$ represents the portion of the system that is not directly influenced. By computing the inverse of this matrix, the method accumulates the effects of repeated interactions between factors. Multiplying Y with this inverse produces matrix T, where each element $t_{ij}$ represents the overall influence of factor i on factor j through all possible paths in the system.

Establishing the α-threshold represents a fundamental phase within the DEMATEL framework [45,46], serving to distinguish substantial causal connections from those with minor impact. In the present research, this benchmark was identified by computing the arithmetic mean of every element contained in the total influence matrix (T). This procedure is a recognized convention in the literature, providing an unbiased filter that ensures only interactions surpassing the mean total effect are preserved for further investigation. Accordingly, any dependency within the T matrix exhibiting a value higher than the α-threshold is classified as a significant component in the system. By excluding lower-intensity correlations that could introduce statistical noise into the evaluation, this approach enhances the integrity and transparency of the resulting causal diagram. Such a method facilitates a concentrated assessment of the primary interlinkages existing throughout the modeled system. This approach was selected because it provides a balanced reference value based directly on the overall intensity of the modeled system interactions and is frequently applied in DEMATEL studies to filter secondary relationships while preserving the main influence structure. To calculate the requisite significance threshold (α) for these interactions, the following mathematical expression is applied:

(5) $$\alpha =\frac{\sum_{i=1}^{n}\sum_{j=1}^{n}\left[t_{ij}\right]}{N}$$

In this context, $t_{ij}$ represents the specific components found within the total influence matrix T, while N indicates the overall number of variables examined throughout the system. Since the study focuses on 8 factors, the matrix contains a total of $N=n^{2}$ elements, which equals 64. Therefore, the calculated threshold value was α=0.399. Only relationships with values higher than this threshold were retained in the causal diagram.

The threshold value α does not influence the computation of the DEMATEL core indicators, including $D_{i}$, $R_{j}$, $D_{i}+R_{j}$, $D_{i}-R_{j}$, and the MICMAC classification. Its role is limited to filtering and visualizing significant relationships in the causal diagram, in order to improve interpretability of the influence structure [47].

In this study, α is used strictly as a visualization parameter applied to the total relation matrix, without affecting the underlying analytical results.

In practical applications, the selection of α may be adapted depending on the decision context and the level of intervention accessibility associated with the system under analysis. For instance, in operational environments where certain system levers are easier to influence, a lower or higher threshold may be selected to emphasize either broader interaction patterns or only the most actionable relationships.

To interpret the role of each factor, two indicators are calculated from matrix T. The first indicator, denoted as $D_{i}$, represents the total influence exerted by factor i on the other factors. It is obtained by summing all elements in the corresponding row of matrix T:

(6) $$D_{i}=\;{\left[\sum_{i=1}^{n}t_{ij}\right]}_{nx1}={\left[t_{i}\right]}_{{nx1}^{\prime }}$$

The second indicator, denoted as $R_{j}$, represents the total influence received by factor j from all other factors. It is calculated by summing the elements in the corresponding column:

(7) $$R_{j}=\;{\left[\sum_{j=1}^{n}t_{ij}\right]}_{1xn}={\left[t_{j}\right]}_{{nx1}^{\prime }}$$

The sum $D_{i}+R_{j}$ indicates the overall involvement of a factor in the system, showing how strongly it is connected to the others. The difference $D_{i}-R_{j}$ indicates whether the factor primarily acts as a source of influence or as a receiver. Positive values suggest that the factor is a driver, while negative values indicate that the factor is mainly affected by the rest of the system.

Using the values of $D_{i}$ and $R_{j}$, the factors are plotted on a two-dimensional diagram. The horizontal axis represents $D_{i}+R_{j}$, which shows the overall importance of each factor. The vertical axis represents $D_{i}-R_{j}$, which separates causal factors from effect factors. This graphical representation provides a clear overview of the structure of relationships between the clinical performance factors of wearable posture monitoring systems and supports the identification of priority areas for system development and clinical implementation.

After completing the DEMATEL calculations and obtaining the total relation matrix T, the study applies the MICMAC method as the next analytical step. MICMAC was chosen because it uses the same total relation matrix already produced by DEMATEL to classify the eight clinical performance factors according to their driving power and dependence power. This classification shows which factors act as the strongest levers for change and which ones are mostly outcomes of the system.

The hybrid DEMATEL–MICMAC sequence was selected for two reasons. First, DEMATEL supplies the complete set of influence values needed for MICMAC. MICMAC starts from the total relation matrix T produced by DEMATEL. Second, DEMATEL identifies the direction of influences, while MICMAC organises the factors into clear priority groups. The serial order of the two methods therefore allows a logical progression from mapping causal links to grouping the factors for practical design and management decisions.

The average driving power $\bar{D}$ and the average dependence $\bar{R}$ are then calculated across all factors. These two averages serve as the reference lines that divide the MICMAC diagram into four quadrants. Each factor is plotted with its driving power on the vertical axis and its dependence power on the horizontal axis. The position of the factor relative to the two averages determines its group and its role in the system:

- Quadrant I (Autonomous factors): low driving power and low dependence. These factors have only weak connections to the rest of the system and can usually be treated as secondary.
- Quadrant II (Relay factors): high driving power and high dependence. These factors both influence and are influenced by many others, so they act as connectors that transmit changes through the network.
- Quadrant III (Output factors): low driving power and high dependence. These factors are mainly outcomes of the system and are strongly affected by upstream variables.
- Quadrant IV (Driving factors): high driving power and low dependence. These factors exert strong influence while depending little on the others, so they represent the primary levers that managers and designers should address first.

This quadrant classification, derived directly from the total relation matrix T completes the structural analysis and provides a practical map for prioritising development efforts in wearable posture monitoring systems.

## 4. Results

The DEMATEL method was applied to analyse the relationships among the critical factors influencing the clinical performance and usability of wearable systems for posture monitoring. The analysis was based on the evaluations provided by 11 experts with professional experience in the development of wearable biomedical devices, covering activities such as hardware design, sensor integration, algorithm development, and clinical documentation. All experts had at least 10 years of professional experience in biomedical engineering and related fields.

The first stage of the results analysis consisted of constructing the normalized direct relation matrix (Table 2), which reflects the average influence scores assigned by the experts after the normalization process described in the methodology section. This matrix contains only direct influences between factors and represents the starting point for calculating indirect and total effects.

Following the normalization step, the total relation matrix was calculated to capture both direct and indirect influences between the factors. This matrix reflects the overall effect that each factor exerts on another when all interaction pathways in the system are considered. The total relation matrix is presented in Table 3.

The values in this matrix represent the accumulated influence of each factor on the others, integrating the cascading effects that occur through intermediate factors.

To distinguish meaningful influence relationships from minor interactions, a threshold value α was calculated based on the average of all elements in the total relation matrix. Only relationships with influence values greater than this threshold were retained for graphical representation and further analysis.

To evaluate the role of each factor within the system, the prominence (D+R) and relation (D−R) indicators were computed from the total relation matrix (Table 4). The value D represents the total influence exerted by a factor on the others, while R represents the total influence received from the others.

The structural interdependencies and the position of each factor are synthesized in a combined causal representation. Based on the values retained after applying the α-threshold, the influence relationships are depicted through a directed network where each factor acts as a node. The connections between these nodes illustrate the direction and intensity of the interaction, with thicker arrows representing relationships that exceed the threshold value.

**Figure 1:**
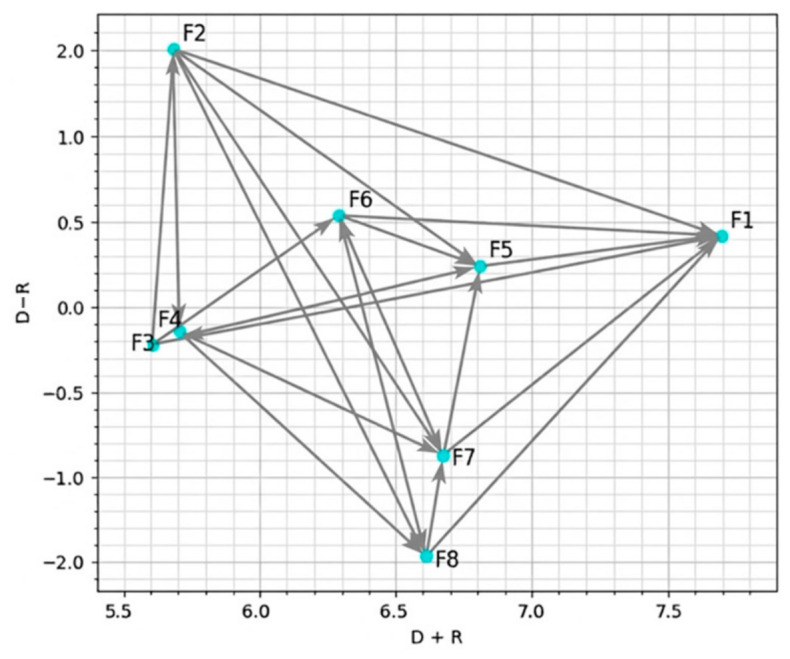
Cause–effect diagram. F_1_ = ability to detect postural deviations; F_2_ = sensitivity to small movement changes; F_3_ = suitability for home monitoring; F_4_ = compliance with standardized measurement methods; F_5_ = real-time feedback capability; F_6_ = continuous monitoring capability; F_7_ = clinical relevance of extracted parameters; F_8_ = capability to assess patient progress over time.

This graphical analysis is further refined by plotting the factors on a two-dimensional cause-effect diagram (Figure 1). The horizontal axis measures the prominence ($D_{i}+R_{j}$), indicating the overall importance and involvement of the factor in the system. The vertical axis represents the net causal influence ($D_{i}-R_{j}$), enabling a clear visualization of whether a factor functions as a primary driver or a resulting effect. The arrows represent relationships of influence (extending from one factor toward the factor being influenced. This integrated visual approach provides a comprehensive overview of the essential interdependencies within the wearable posture monitoring system.

**Figure 2:**
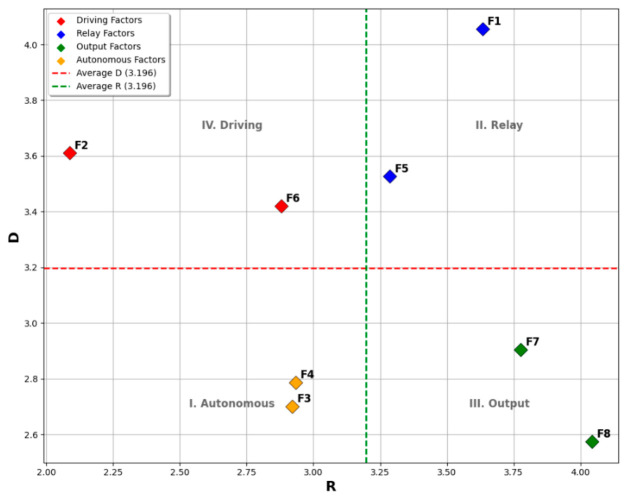
MICMAC diagram. F_1_ = ability to detect postural deviations; F_2_ = sensitivity to small movement changes; F_3_ = suitability for home monitoring; F_4_ = compliance with standardized measurement methods; F_5_ = real-time feedback capability; F_6_ = continuous monitoring capability; F_7_ = clinical relevance of extracted parameters; F_8_ = capability to assess patient progress over time.

Based on the calculated driving power $D_{i}$ and dependence power $R_{i}$ for each factor, the MICMAC classification was performed using the average values $\bar{D}=3.19$, and $\bar{R}=3.19$ as reference thresholds. Each factor was compared against these averages to determine its position within the four MICMAC quadrants, namely autonomous (I), relay (II), outputs (III), and drivers (IV). This classification enables a clear identification of the role played by each factor within the system, distinguishing between key driving variables, outcome variables, and intermediary elements. The results of this classification are presented in Figure 2.

The MICMAC diagram places F_2_ and F_6_ in the determinant group. These factors exert strong influence while depending little on the others. F_1_ and F_5_ fall into the relay group and act as connectors that both affect and receive influence from several variables. F_7_ and F_8_ appear as dependent factors. F_3_ and F_4_ are autonomous and sit outside the main network of interactions. This grouping matches the cause–effect diagram from DEMATEL and shows the same priority order for design and management decisions.

## 5. Discussion

The DEMATEL results map out a straightforward set of relationships among the eight clinical performance factors for wearable posture monitoring systems. The cause group consists of F_1_ (ability to detect postural deviations), F_2_ (sensitivity to small movement changes), F_5_ (real-time feedback capability), and F_6_ (continuous monitoring capability). These factors show positive $D_{i}-R_{j}$ values, which means they push influence outward through the network. F_2_ records the highest net cause value at 1.520, so it functions as the clearest starting point for change. F_1_ shows the highest prominence score of 7.690, which indicates a central position in the system through both incoming and outgoing links, without implying a purely driving role. The effect group includes F_3_ (suitability for home monitoring), F_4_ (compliance with standardized measurement methods), F_7_ (clinical relevance of extracted parameters), and F_8_ (capability to assess patient progress over time). Their negative $D_{i}-R_{j}$ values indicate they absorb influence rather than generate it, with F_8_ showing the strongest net effect at −1.470. F_7_ and F_8_ also carry high prominence scores, which place them as central outcomes that reflect the overall health of the system.

The integration of the MICMAC analysis refines this causal structure by grouping the same factors according to their driving and dependence powers. Using the average thresholds, the factors are distributed into four distinct categories that clarify their functional role within the system. F_2_ and F_6_ are positioned in the determinant quadrant (IV), confirming that they act as the primary driving forces with strong influence and low dependence. This classification follows the driving and dependence values in Table 4, where determinant status is defined by high $D_{i}-R_{j}$ and low dependence, rather than by prominence alone. F_1_ and F_5_ fall into the relay quadrant (II), indicating that they both influence and are influenced by other factors. Their position highlights their role as transmission nodes that propagate the effects generated by determinant factors toward the rest of the system.

The effect group identified through DEMATEL is further differentiated by MICMAC. F_7_ and F_8_ are classified as output factors (III), which confirms their role as final outcomes that accumulate the effects of upstream variables. Their high dependence values explain why improvements in determinant and relay factors directly translate into enhanced clinical relevance and a better assessment of patient progress. In contrast, F_3_ and F_4_ are placed in the autonomous quadrant (I), showing low driving and low dependence. This positioning indicates that, although they are part of the system, their influence on overall performance is limited compared to the other factors.

The combined DEMATEL–MICMAC results therefore provide a more structured interpretation of the system. DEMATEL identifies the direction and intensity of influence, while MICMAC clarifies the hierarchical positioning of factors in terms of strategic importance. Together, they confirm that sensitivity to minor motion changes (F_2_) and continuous monitoring capability (F_6_) represent the main entry points for intervention, while postural deviation detection (F_1_) and real-time feedback (F_5_) serve as critical intermediaries that transfer these improvements toward the outcome layer.

Looking at the cause–effect diagram, the horizontal axis ($D_{i}+R_{j}$) spreads the factors from left to right according to how deeply they sit inside the network. F_1_ has the highest prominence score, which places it toward the right side of the diagram as a highly connected node rather than a pure driver. F_2_ sits higher on the vertical axis but farther left, confirming its role as a pure driver with less overall centrality than F_1_. The effect factors cluster lower on the vertical axis and spread across the middle to right of the prominence scale. Arrows in the underlying total relation matrix flow heavily from the cause cluster toward F_7_ and F_8_, with F_1_ and F_2_ sending the thickest connections. F_5_ and F_6_ sit in between, passing influence forward after they receive inputs from F_1_ and F_2_. This pattern holds across the matrix entries above the α-threshold: direct and indirect paths converge on repeatability and comfort as the final receivers.

Sensitivity to minor motion changes (F_2_) emerges as the factor that deserves first attention in any design or improvement effort. Its high net cause value means that even modest gains in sensitivity to minor motion changes ripple outward to nearly every other element. Recent work [48,49,50] on inertial measurement units in rehabilitation has reached the same conclusion. For example, a study published in 2025 shows that sensors with higher resolution for small angular shifts improve detection of early compensatory patterns in stroke patients and older adults recovering from hip fractures [48]. The meta-analysis reported statistically significant improvements in gait speed (SMD = 0.41, *p* = 0.02) and allowed earlier identification of gait asymmetry compared with conventional observation [48]. These findings line up with the DEMATEL placement of F_2_ at the top of the cause group. When sensitivity improves, the system gains the raw data needed for F_1_ to operate accurately, which then feeds into F_5_ and F_6_. The chain continues to F_7_ and F_8_ without requiring separate interventions at the outcome level.

Postural deviation detection capability (F_1_) acts as the network hub. Its prominence score reflects the volume of connections it maintains in both directions. In the total relation matrix, F_1_ records the largest row sums for outgoing influence and still receives noticeable incoming effects from F_2_ and F_6_. This central position matches reports from the last five years on wearable systems for postural control [48,51]. Researchers testing IMU-based posture trackers in home settings noted that detection accuracy for deviations larger than 5° served as the single best predictor of overall device utility across multiple clinical trials. The same meta-analysis showed that reliable deviation alerts produced significant gains in balance (Berg Balance Scale, SMD = 0.44, *p* = 0.03) and functional mobility (Timed Up and Go Test, SMD = −0.36, *p* = 0.01) [48]. In the present analysis, F_1_ occupies exactly that mediating role: it translates upstream sensitivity gains into measurable effects on clinical relevance and progress tracking.

Real-time feedback capability (F_5_) and continuous monitoring capability (F_6_) function as practical bridges within the cause group. Real-time feedback (F_5_) sits close to the center of the prominence scale with a modest positive relation value. Continuous monitoring capability (F_6_) shows a stronger net cause score than F_5_, which suggests that maintaining an uninterrupted stream of data. Literature from 2025 to 2026 supports this intermediate position [48,49]. The meta-analysis confirmed that real-time auditory or haptic cues delivered during extended monitoring periods improved functional mobility scores more than delayed feedback [48]. These external results echo the DEMATEL arrows that run from F_6_ to F_5_ and onward to the effect cluster. The ability to monitor continuously and provide feedback together converts raw detection and sensitivity into usable clinical signals without adding burden to the user.

On the effect side, capability to assess patient progress over time (F_8_) records the most negative relation value and a high prominence score. The longitudinal assessment of progress therefore acts as the final indicator of system success. Any weakness here absorbs and amplifies shortcomings from the cause factors. From an economic perspective, this position is critical, as a limited ability to track recovery directly translates into reduced clinical utility, shorter usage cycles, and ultimately lower return on investment for both healthcare providers and patients. A recent quantitative study [52] on continued wearable use among older adults confirm this pattern. One survey of community-dwelling participants over 65 found that comfort ranked ahead of battery life and data accuracy as the strongest predictor of device retention after three months [52]. The same study linked comfort directly to adherence during unsupervised home exercises, which aligns with F_8_’s position as the lowest point on the cause–effect diagram. In economic terms, higher adherence driven by comfort improves cost-effectiveness by maximizing the therapeutic value extracted from each deployed device and reducing the need for additional clinical interventions. F_7_ (clinical relevance of extracted parameters) sits just above F_8_ in the effect group. Its negative relation value and solid prominence indicate that repeatability depends on stable inputs from F_1_, F_2_, and F_6_.

Suitability for home monitoring (F_3_) and compliance with standardized measurement methods (F_4_) occupy the effect group with smaller negative relation values, which suggests they respond to cause-factor changes but do not dominate the outcome layer. Suitability for home monitoring (F_3_) and compliance with standardized methods (F_4_) therefore emerge as secondary consequences rather than primary levers. This ordering fits findings from 2024 and 2025 evaluations of remote rehabilitation platforms [53,54]. Teams deploying smart vests with embedded sensors for hip-fracture recovery observed that home suitability scores rose only after detection accuracy and feedback latency improved in controlled testing [53]. Similarly, studies comparing wearable outputs against laboratory goniometers reported that alignment with clinical standards (F_4_) depended on upstream sensor performance rather than on device firmware alone [54]. The DEMATEL diagram places these two factors left of center on the prominence axis and below the zero line on the relation axis, which matches the external evidence that they follow rather than lead the system. From an economic standpoint, this positioning suggests that investments directed prematurely toward deployment scalability or regulatory alignment may yield limited returns unless core technical performance is first optimized.

Taken together, the structure points to a clear management priority: allocate resources first to F_2_ and F_1_ toward high-impact technical drivers that maximize both clinical and economic efficiency across the system lifecycle. Gains here lift the entire network, including the high-prominence effect factors F_7_ and F_8_. Designers can use this ordering when specifying hardware requirements or selecting component suppliers. For instance, choosing IMUs with sub-degree resolution and stable drift characteristics directly addresses F_2_, which then strengthens F_1_ without separate development budgets for feedback modules, comfort materials or long-term data storage solutions. Clinicians evaluating devices for postural rehabilitation programs can apply the same hierarchy during procurement. A system that scores well on sensitivity and deviation detection will likely deliver a clear picture of patient progress even if its initial home-monitoring interface looks basic. This approach reduces trial-and-error cycles in pilot deployments and shortens the time needed to reach measurable patient progress.

The analysis also carries implications for training and implementation. Rehabilitation teams can focus initial patient education on the two causal factors that patients can influence indirectly: ensuring device up-time for continuous monitoring (F_6_) and consistent wear to preserve sensitivity (F_2_). Once these are in place, real-time feedback (F_5_) becomes more effective, and the downstream benefits in clinical relevance and progress assessment. This sequence mirrors protocols tested in recent biofeedback locomotion studies, where therapists first verified sensor positioning and sensitivity thresholds before activating auditory cues [48]. The result was faster gains in functional mobility compared with protocols that introduced feedback without prior stability checks.

Comparisons with other DEMATEL applications in wearable and rehabilitation contexts reinforce the present findings. A 2025 study that combined fuzzy Delphi and DEMATEL to prioritize design indicators for sustainable health-management wearables identified user-centric outcomes such as long-term adherence and progress tracking as net effect factors, while sensor performance metrics sat firmly in the cause group [55]. Another 2024 investigation applied fuzzy-DEMATEL inside a smart product-service system for dynamic rehabilitation management and reached the same causal ordering: data-acquisition capabilities drove service-level outcomes. A 2024 evaluation of smart rehabilitation systems using DEMATEL listed motion-detection accuracy and sensor responsiveness as primary drivers, with home suitability and user comfort listed as dependent results [51]. A 2025 evaluation of smart monitoring systems in elderly care facilities used DEMATEL alongside ANP and listed motion-detection accuracy and sensor responsiveness as primary drivers, with home suitability and longitudinal assessment listed as dependent results [54]. Across these independent projects, the same directional pattern appears despite differences in exact factor wording or expert panels. The consistency suggests that the underlying network structure for wearable clinical performance is stable across related domains.

This study stands out because it applies the DEMATEL method for the first time to map the exact clinical performance factors that decide whether a wearable posture-monitoring device actually helps patients in everyday rehabilitation. Most earlier papers tested single devices for accuracy or asked users about satisfaction in separate surveys. Here the analysis treats all eight factors as parts of one connected system and shows which ones drive the others. The resulting cause–effect diagram gives designers and clinicians a practical order of priorities instead of a simple list of features. This network-based perspective represents a new analytical approach in the specific context of postural rehabilitation, where previous studies have not modeled the directional relationships between clinical performance factors.

Future studies could enlarge the expert panel to include practicing physiotherapists, rehabilitation physicians, and patients who have worn the devices in home settings over extended periods. Their direct-relation scores would help determine whether the current cause–effect structure remains stable when end-user perspectives are incorporated. Prototype testing over longer time spans would also allow researchers to verify whether improvements in the identified cause factors lead to measurable gains data relevance, assessment accuracy, and adherence under real daily conditions. An additional line of research could involve combining DEMATEL with complementary decision-making tools such as the ANP to quantify the strength of interdependencies and to build a hybrid evaluation framework that links expert judgment with empirical performance data. Applying the same analytical framework to devices intended for other rehabilitation groups, such as stroke survivors or children with scoliosis, would further clarify how transferable the current factor network is across clinical populations.

In summary, the DEMATEL diagram and numerical indicators offer a practical roadmap for both designers and clinicians. Sensitivity to small movement changes and postural deviation detection capability sit at the head of the influence chain. Improvements here propagate through real-time feedback and continuous monitoring to reach the key outcomes of clinical relevance and patient progress assessment. F_1_ is positioned as a central connecting element due to its high prominence, while F_2_ retains the role of primary driving factor within the system. The MICMAC classification reinforces this structure by showing that F_2_ and F_6_ act as determinant factors with high driving power and low dependence, while F_1_ and F_5_ function as relay factors that transfer influence across the system. The dependent positioning of F_7_ and F_8_ further confirms their role as outcome indicators, whereas F_3_ and F_4_ remain autonomous with limited impact on system-wide performance. By focusing development and selection efforts on the main cause and determinant factors identified in this study, teams can increase the likelihood that technical improvements will translate into observable clinical benefits. This structured prioritization offers a concrete bridge between engineering design choices and rehabilitation outcomes, which has been only loosely addressed in earlier wearable-sensor studies.

## 6. Conclusions

The DEMATEL analysis identifies sensitivity to small movement changes (F_2_) and postural deviation detection capability (F_1_) as the main drivers of the entire system. Real-time feedback (F_5_) and continuous monitoring capability (F_6_) act as useful bridges that carry influence forward. On the receiving side, clinical relevance of extracted parameters (F_7_) and capability to assess patient progress over time (F_8_) emerge as the final outcomes that reflect the quality of the whole device. Suitability for home monitoring (F_3_) and compliance with standardized measurement methods (F_4_) follow rather than lead the other factors. This causal ordering is further supported by the MICMAC classification, where F_2_ and F_6_ are identified as determinant factors (high driving, low dependence), F_1_ and F_5_ as relay factors, F_7_ and F_8_ as dependent factors, and F_3_ and F_4_ as autonomous variables. This clear ordering means that any effort to raise sensor resolution or detection accuracy will lift the rest of the network, including the two high-prominence outcomes that matter most to patients and therapists.

The findings provide device manufacturers with a concise set of priorities during component selection, system architecture design, and specification drafting. By directing resources toward the identified cause factors, development teams can achieve broader performance improvements without dispersing effort across less influential features. Clinicians responsible for selecting wearable devices for postural rehabilitation programs can apply the same hierarchy during procurement and clinical integration, starting with an evaluation of sensitivity and deviation detection, followed by an assessment of feedback clarity and calibration simplicity for home users. The MICMAC results strengthen this prioritization by clearly separating primary drivers from dependent outcomes, enabling more confident decision-making regarding resource allocation and system design. This shared priority structure supports closer alignment between technical evaluation and clinical decision-making, reducing the gap that often exists between engineering specifications and therapeutic expectations.

Several limitations should be considered when interpreting the results. The expert panel consisted of eleven professionals, all of whom were drawn from engineering and device-development roles. Although these experts possessed the required experience, their professional background may have influenced the way they assessed the importance and relationships of the performance factors. Engineers often focus on signal quality, detection precision, and hardware reliability, while frontline therapists and long-term users tend to place greater emphasis on comfort, usability, and adherence during daily routines. As a result, the direct-relation matrix is based on a relatively narrow professional perspective, and the causal structure of the network might change if clinicians, ergonomics specialists, and patients were included in the evaluation process. In addition, although MICMAC enhances the interpretation of factor roles, its results remain dependent on the DEMATEL-derived matrix, and therefore inherit the same expert-based subjectivity.

The factor set was also defined specifically for adult postural rehabilitation. A different configuration of relationships might emerge in pediatric populations or in patients with neurological conditions such as Parkinson’s disease, where movement patterns and therapy goals differ substantially. In addition, the present study relies entirely on expert estimates and does not incorporate longitudinal data collected from patients using wearable prototypes in real environments. Field trials and long-term monitoring studies would therefore be necessary to confirm that improvements in the identified cause factors lead to measurable clinical progress and sustained device use outside controlled settings. Future research could also extend the current DEMATEL–MICMAC framework by integrating quantitative validation methods, such as ANP or empirical performance data, to further strengthen the robustness of the classification.

From a biomimetic perspective, the causal structure can be interpreted as a layered response system, where low-level sensory inputs influence higher-level functional outcomes in a stepwise manner. This supports the idea that wearable systems can be designed as adaptive networks rather than isolated functional modules.

Despite these constraints, the study provides a structured map of interdependencies that has been largely absent from earlier research focused mainly on isolated validation tests. Designers and project managers can use the identified causal hierarchy to set development targets in a more focused manner, while rehabilitation teams gain a clearer framework for selecting and integrating wearable systems into therapy programs. The combined DEMATEL–MICMAC approach offers both causal insight and hierarchical classification, improving the practical applicability of the results. The cause–effect diagram and its numerical indicators can be updated whenever new sensor technologies or clinical protocols emerge, allowing the prioritization model to evolve alongside technological progress. By framing wearable posture monitoring as a system of interacting clinical and technical factors, the study introduces a decision-support perspective that can be reused in future evaluations and extended to other forms of digital rehabilitation equipment. From an economic standpoint, this approach supports more efficient allocation of development resources by directing investment toward features that generate the strongest downstream benefits, which contributes to improved scalability and long-term sustainability of wearable rehabilitation solutions in real healthcare environments.

## Figures and Tables

**Table 1:** Factors included in the DEMATEL analysis.

Symbol	Factor	References
F_1_	Postural deviation detection capability	[1,10,18]
F_2_	Sensitivity to minor motion changes	[3,4,7]
F_3_	Suitability for home monitoring	[8,9,30]
F_4_	Compliance with standardized measurement methods	[4,6,12]
F_5_	Real-time feedback capability	[5,26,27,42,43]
F_6_	Continuous monitoring capability	[5,8,15]
F_7_	Clinical relevance of extracted parameters	[2,13,19]
F_8_	Capability to assess patient progress over time	[8,15,28]

**Table 2:** Normalised direct relations matrix.

Factor	F_1_	F_2_	F_3_	F_4_	F_5_	F_6_	F_7_	F_8_
F_1_	0.000	0.107	0.093	0.147	0.178	0.116	0.178	0.178
F_2_	0.142	0.000	0.084	0.133	0.138	0.080	0.147	0.142
F_3_	0.107	0.022	0.000	0.080	0.102	0.107	0.093	0.125
F_4_	0.125	0.080	0.053	0.000	0.075	0.080	0.125	0.111
F_5_	0.125	0.067	0.111	0.120	0.000	0.142	0.147	0.142
F_6_	0.174	0.080	0.107	0.040	0.133	0.000	0.125	0.147
F_7_	0.125	0.067	0.102	0.102	0.084	0.071	0.000	0.138
F_8_	0.093	0.053	0.125	0.071	0.080	0.080	0.102	0.000

F_1_ = ability to detect postural deviations; F_2_ = sensitivity to small movement changes; F_3_ = suitability for home monitoring; F_4_ = compliance with standardized measurement methods; F_5_ = real-time feedback capability; F_6_ = continuous monitoring capability; F_7_ = clinical relevance of extracted parameters; F_8_ = capability to assess patient progress over time.

**Table 3:** Total influence matrix highlighting (*) factors with significant influence (α=0.399).

Factor	F_1_	F_2_	F_3_	F_4_	F_5_	F_6_	F_7_	F_8_
F_1_	0.451 (*)	0.356	0.451 (*)	0.494 (*)	0.559 (*)	0.462 (*)	0.623 (*)	0.656 (*)
F_2_	0.526 (*)	0.232	0.402 (*)	0.446 (*)	0.485 (*)	0.393	0.548 (*)	0.573 (*)
F_3_	0.396	0.197	0.244	0.315	0.364	0.337	0.397	0.446 (*)
F_4_	0.421 (*)	0.254	0.302	0.252	0.352	0.319	0.435 (*)	0.447 (*)
F_5_	0.504 (*)	0.289	0.418 (*)	0.422 (*)	0.354	0.437	0.536 (*)	0.563 (*)
F_6_	0.530 (*)	0.294	0.407 (*)	0.351	0.466 (*)	0.304	0.509 (*)	0.556 (*)
F_7_	0.432 (*)	0.249	0.354	0.354	0.370	0.323	0.336	0.482 (*)
F_8_	0.371	0.214	0.343	0.297	0.333	0.302	0.389	0.319

F_1_ = ability to detect postural deviations; F_2_ = sensitivity to small movement changes; F_3_ = suitability for home monitoring; F_4_ = compliance with standardized measurement methods; F_5_ = real-time feedback capability; F_6_ = continuous monitoring capability; F_7_ = clinical relevance of extracted parameters; F_8_ = capability to assess patient progress over time.

**Table 4:** Prominence and relation values of the factors.

Factor	$D_{i}$	$R_{j}$	$D_{i}+R_{j}$	$D_{i}-R_{j}$	Dominant Characteristic
F_1_	4.055	3.635	7.690	0.419	Cause
F_2_	3.610	2.089	5.699	1.520	Cause
F_3_	2.699	2.922	5.622	−0.223	Effect
F_4_	2.785	2.935	5.721	−0.149	Effect
F_5_	3.526	3.287	6.813	0.239	Cause
F_6_	3.419	2.881	6.301	0.537	Cause
F_7_	2.903	3.777	6.680	−0.873	Effect
F_8_	2.573	4.044	6.617	−1.470	Effect

F_1_ = ability to detect postural deviations; F_2_ = sensitivity to small movement changes; F_3_ = suitability for home monitoring; F_4_ = compliance with standardized measurement methods; F_5_ = real-time feedback capability; F_6_ = continuous monitoring capability; F_7_ = clinical relevance of extracted parameters; F_8_ = capability to assess patient progress over time.

## Data Availability

The data that support the findings of this study are available from the corresponding author upon reasonable request.

## Appendix A

The raw data from the direct influence matrix are presented in this appendix to support the consistency of the findings and to allow for an independent verification of the results.

**Table A1:** Direct influence matrix.

Factor	F_1_	F_2_	F_3_	F_4_	F_5_	F_6_	F_7_	F_8_
F_1_	0.000	2.182	1.909	3.000	3.636	2.364	3.636	3.636
F_2_	2.909	0.000	1.727	2.727	2.818	1.636	3.000	2.909
F_3_	2.182	0.455	0.000	1.636	2.091	2.182	1.909	2.545
F_4_	2.545	1.636	1.091	0.000	1.545	1.636	2.545	2.273
F_5_	2.545	1.364	2.273	2.455	0.000	2.909	3.000	2.909
F_6_	3.545	1.636	2.182	0.818	2.727	0.000	2.545	3.000
F_7_	2.545	1.364	2.091	2.091	1.727	1.455	0.000	2.818
F_8_	1.909	1.091	2.545	1.455	1.636	1.636	2.091	0.000

F_1_ = ability to detect postural deviations; F_2_ = sensitivity to small movement changes; F_3_ = suitability for home monitoring; F_4_ = compliance with standardized measurement methods; F_5_ = real-time feedback capability; F_6_ = continuous monitoring capability; F_7_ = clinical relevance of extracted parameters; F_8_ = capability to assess patient progress over time.

## Appendix B

Appendix B presents the standard deviation (SD) values associated with the direct relation matrix used in the DEMATEL analysis (Table A2). The calculations were performed using the evaluations provided by the 11 experts included in the study. Each value reflects the variability of expert judgments regarding the influence exerted by one factor on another. Overall, the results indicate moderate dispersion across most relationships, suggesting an acceptable level of consistency among expert assessments. Higher deviation values were observed mainly for relationships involving subjective evaluations related to monitoring conditions and interpretation of clinical relevance, while lower values appeared in relationships associated with well-established technical functionalities.

**Table A2:** Standard deviation values associated with the direct relation.

Factor	F_1_	F_2_	F_3_	F_4_	F_5_	F_6_	F_7_	F_8_
F_1_	0.000	1.537	0.944	1.095	0.674	1.804	0.505	0.467
F_2_	1.136	0.000	1.009	0.905	1.328	1.502	1.342	1.375
F_3_	1.662	0.82	0.000	1.286	1.471	1.601	1.514	1.368
F_4_	1.508	1.362	1.221	0.000	0.905	1.286	1.44	1.009
F_5_	1.753	1.69	1.348	1.44	0.000	1.079	1.265	1.3
F_6_	0.688	1.502	1.328	1.079	1.286	0.000	1.508	1.549
F_7_	1.572	1.206	1.136	1.578	1.421	1.368	0.000	1.471
F_8_	1.578	1.578	1.214	1.368	1.120	1.690	1.578	0.000

F_1_ = ability to detect postural deviations; F_2_ = sensitivity to small movement changes; F_3_ = suitability for home monitoring; F_4_ = compliance with standardized measurement methods; F_5_ = real-time feedback capability; F_6_ = continuous monitoring capability; F_7_ = clinical relevance of extracted parameters; F_8_ = capability to assess patient progress over time.

The SD values indicated moderate dispersion across the evaluated relationships. Considering that the assessment scale ranged from 0 to 4, the observed variability suggests an acceptable level of consistency among expert judgments. Lower deviation values were mainly associated with relationships linked to established technical functions, while higher dispersion appeared in evaluations involving clinically interpretative aspects and long-term monitoring considerations.

In this study, the SD of experts’ responses was calculated for each element of the initial direct influence matrix. Unlike many DEMATEL studies that report only the overall mean SD or aggregated indicators, the present research performed a detailed cell-by-cell analysis. The mean standard deviation across all pairs was 1.137, with 72% of the values being lower than 1.5. Given the complex nature of the investigated topic and the 0–4 evaluation scale used, these values indicate an acceptable level of consensus among experts, consistent with practices found in the literature [56]. The higher SD values reflect the inherent subjectivity in assessing influence relationships within this domain.

To evaluate the reliability of the results obtained, a sensitivity analysis was conducted by adjusting the α-threshold (Table A3). This step involved increasing and decreasing the threshold value by 10% to observe the impact on the causal relationships represented in Figure 1. This procedure helps in identifying how stable the connections between factors remain when the selection criteria undergo minor changes.

**Table A3:** Sensitivity analysis for the cause–effect diagram.

Factor	F_1_	F_2_	F_3_	F_4_	F_5_	F_6_	F_7_	F_8_
F_1_								
F_2_								
F_3_								
F_4_								
F_5_								
F_6_								
F_7_								
F_8_								

F_1_ = ability to detect postural deviations; F_2_ = sensitivity to small movement changes; F_3_ = suitability for home monitoring; F_4_ = compliance with standardized measurement methods; F_5_ = real-time feedback capability; F_6_ = continuous monitoring capability; F_7_ = clinical relevance of extracted parameters; F_8_ = capability to assess patient progress over time.

Table A3 presents the results of this analysis. The cells marked with green represent the relationships from the initial model that remain constant in both the −10% and +10% scenarios. The blue cells indicate the connections that are removed from the diagram when the α-threshold is increased by 10%. The orange cells highlight the new relationships that appear only when the threshold is lowered by 10%.

The sensitivity analysis highlights the stability of the causal model. The presence of a large number of green cells indicates that the core structure of the network is solid. These primary relationships remain unchanged even when the filtering criteria are modified by 10% in either direction, which suggests that the identified interdependencies are consistent.

The connections marked in blue disappear when the α-threshold is increased. This change shows that those specific links are weaker and depend on a more permissive threshold to be included in the diagram. On the other hand, the orange cells represent secondary relationships. These emerge only when the threshold is lowered, indicating that they are less significant in the overall structure of the model. This differentiation allows for a clear distinction between the essential causal paths and the peripheral ones, ensuring that the conclusions are based on the most stable interactions within the system.

## References

[1] Porciuncula F., Roto A.V., Kumar D., Davis I., Roy S., Walsh C.J., Awad L.N. (2018). Wearable Movement Sensors for Rehabilitation: A Focused Review of Technological and Clinical Advances. PM&R.

[2] Wang Q., Markopoulos P., Yu B., Chen W., Timmermans A. (2017). Interactive Wearable Systems for Upper Body Rehabilitation: A Systematic Review. J. Neuroeng. Rehabil..

[3] Alemayoh T.T., Lee J.H., Okamoto S. (2023). Leg Joint Angle Estimation from a Single Inertial Sensor During Variety of Walking Motions: A Deep Learning Approach. IEEE Access.

[4] Horenstein R.E., Lewis C.L., Yan S., Halverstadt A., Shefelbine S.J. (2019). Validation of Magneto-Inertial Measuring Units for Measuring Hip Joint Angles. J. Biomech..

[5] Xu C., Tan Y., Strout Z., Liu G., Zhu K., Wang H., Shull P.B. (2026). Real-Time OpenSim via IMUs for Full Body Kinematics During Gait, Sports, Exercise, and Dance Movements. IEEE Trans. Neural Syst. Rehabil. Eng..

[6] Piche E., Guilbot M., Chorin F., Guerin O., Zory R., Gerus P. (2022). Validity and Repeatability of a New Inertial Measurement Unit System for Gait Analysis on Kinematic Parameters: Comparison with an Optoelectronic System. Measurement.

[7] Oliveira N., Park J., Barrance P. (2023). Using Inertial Measurement Unit Sensor Single Axis Rotation Angles for Knee and Hip Flexion Angle Calculations During Gait. IEEE J. Transl. Eng. Health Med..

[8] Rodrigues I.B., Tariq S., Kouroukis A., Swance R., Adachi J., Bray S., Fang Q., Ioannidis G., Kobsar D., Rabinovich A. (2024). Mapping Sedentary Behaviour (MAPS-B) in Winter and Spring Using Wearable Sensors, Indoor Positioning Systems, and Diaries in Older Adults Who Are Pre-Frail and Frail: A Feasibility Longitudinal Study. PLoS ONE.

[9] Schönfeldt A., Maylor B., Chen X., Clark R., Doherty A. (2025). Reducing Annotation Burden in Physical Activity Research Using Vision Language Models. Sci. Rep..

[10] Vähä-Ypyä H., Husu P., Suni J., Vasankari T., Sievänen H. (2018). Reliable Recognition of Lying, Sitting, and Standing with a Hip-Worn Accelerometer. Scand. J. Med. Sci. Sports.

[11] Adjel M., Dumas R., Mohammed S., Bonnet V. (2025). Influence of Visual-Inertial Sensor-to-Segment Calibration on Upper Limb Joint Angles Estimation From Multiple Inverse Kinematics Methods. IEEE Trans. Autom. Sci. Eng..

[12] Mallat R., Bonnet V., Dumas R., Adjel M., Venture G., Khalil M., Mohammed S. (2021). Sparse Visual-Inertial Measurement Units Placement for Gait Kinematics Assessment. IEEE Trans. Neural Syst. Rehabil. Eng..

[13] Sengupta N., Rao A.S., Yan B., Palaniswami M. (2024). A Survey of Wearable Sensors and Machine Learning Algorithms for Automated Stroke Rehabilitation. IEEE Access.

[14] Xue Z., Gai Y., Wu Y., Liu Z., Li Z. (2024). Wearable Mechanical and Electrochemical Sensors for Real-Time Health Monitoring. Commun. Mater..

[15] Li J., Zhu K., Li D., Kang P., Shull P.B. (2024). 3D Knee and Hip Angle Estimation with Reduced Wearable IMUs via Transfer Learning During Yoga, Golf, Swimming, Badminton, and Dance. IEEE Trans. Neural Syst. Rehabil. Eng..

[16] Hernandez V., Dadkhah D., Babakeshizadeh V., Kulić D. (2021). Lower Body Kinematics Estimation from Wearable Sensors for Walking and Running: A Deep Learning Approach. Gait Posture.

[17] Neagu M.D., Morega A.M., Mogoş L. (2010). Intratumoral Temperature Monitoring. U.P.B. Sci. Bull. Ser. C.

[18] Montesinos L., Castaldo R., Pecchia L. (2018). Wearable Inertial Sensors for Fall Risk Assessment and Prediction in Older Adults: A Systematic Review and Meta-Analysis. IEEE Trans. Neural Syst. Rehabil. Eng..

[19] Favata A., Gallart-Agut R., Pàmies-Vilà R., Torras C., Font-Llagunes J.M. (2024). IMU-Based Systems for Upper-Limb Kinematic Analysis in Clinical Applications: A Systematic Review. IEEE Sens. J..

[20] Mazilu C., Anghel M. (2021). Intelligent Patient Management for Improving Quality of Medical Services. U.P.B. Sci. Bull. Ser. C.

[21] Drăgoi M.-V., Frimu A.-V., Postelnicu A., Puiu R.-A., Petrea G., Hank A. (2026). Interactive Teleoperation of an Articulated Robotic Arm Using Vision-Based Human Hand Tracking. Biomimetics.

[22] Drăgoi M.-V., Hadăr A., Goga N., Baciu F., Ștefan A., Grigore L.Ș., Gorgoteanu D., Molder C., Oncioiu I. (2023). Contributions to the Dynamic Regime Behavior of a Bionic Leg Prosthesis. Biomimetics.

[23] Badea C.R. (2019). Study on the Influence of Contact Points, Scenarios and Graphical Reference Elements on the Motion Analysis Process, Carried out Using the Inertial Mechatronic System MVN Analyze. Int. J. Mechatron. Appl. Mech..

[24] Zhu C., Luo L., Li R., Guo J., Wang Q. (2024). Wearable Motion Analysis System for Thoracic Spine Mobility with Inertial Sensors. IEEE Trans. Neural Syst. Rehabil. Eng..

[25] Tedesco S., Belcastro M., Torre O.M., Torchia P., Alfieri D., Khokhlova L., O’Flynn B. A Multi-Sensors Wearable System for Remote Assessment of Physiotherapy Exercises during ACL Rehabilitation. Proceedings of the 2019 26th IEEE International Conference on Electronics, Circuits and Systems (ICECS).

[26] Digo E., Gastaldi L., Antonelli M., Pastorelli S., Cereatti A., Caruso M. (2022). Real-Time Estimation of Upper Limbs Kinematics with IMUs during Typical Industrial Gestures. Procedia Comput. Sci..

[27] Bethi S.R., RajKumar A., Vulpi F., Raghavan P., Kapila V. Wearable Inertial Sensors for Exergames and Rehabilitation. Proceedings of the 2020 42nd Annual International Conference of the IEEE Engineering in Medicine & Biology Society (EMBC).

[28] Qiu S., Wang Z., Zhao H., Liu L., Jiang Y. (2018). Using Body-Worn Sensors for Preliminary Rehabilitation Assessment in Stroke Victims With Gait Impairment. IEEE Access.

[29] Routhier F., Duclos N.C., Lacroix É., Lettre J., Turcotte E., Hamel N., Michaud F., Duclos C., Archambault P.S., Bouyer L.J. (2020). Clinicians’ Perspectives on Inertial Measurement Units in Clinical Practice. PLoS ONE.

[30] Proietti T., Bandini A. (2024). Wearable Technologies for Monitoring Upper Extremity Functions During Daily Life in Neurologically Impaired Individuals. IEEE Trans. Neural Syst. Rehabil. Eng..

[31] Klaassen B., van Beijnum B.-J.F., Held J.P., Reenalda J., van Meulen F.B., Veltink P.H., Hermens H.J. (2017). Usability Evaluations of a Wearable Inertial Sensing System and Quality of Movement Metrics for Stroke Survivors by Care Professionals. Front. Bioeng. Biotechnol..

[32] Nicolescu A.F., Coman C.G., Cristoiu C.A. (2017). Calculus Algorithm for Evaluation of Gravitational and Inertial Loads Acting on a Scara Industrial Robot in Pick and Place Applications. Proc. Manuf. Syst..

[33] Drăgoi M.-V., Nisipeanu I., Puiu R.-A., Tache F.-G., Spiridon-Mocioacă T.-M., Hank A., Cristoiu C. (2025). An Inclusive Offline Learning Platform Integrating Gesture Recognition and Local AI Models. Biomimetics.

[34] Liu W.-Y., Tung T.-H., Chuang Y.-C., Chien C.-W. (2021). Using DEMATEL Technique to Identify the Key Success Factors of Shared Decision-Making Based on Influential Network Relationship Perspective. J. Healthc. Eng..

[35] Stanciu A., Țîțu A.M., Hrybiuk O., Machado J., Cioboată D.D. (2024). Industry 4.0. Upsides and Downsides. Towards Industry 5.0. Proceedings of the International Conference on Reliable Systems Engineering (ICoRSE), Bucharest, Romania, 5–6 September 2024.

[36] Liao C.-H., Bercea S. (2021). Success Factors of Health Promotion: Evaluation by DEMATEL and M-DEMATEL Methods—A Case Study in a Non-Profit Organization. PLoS ONE.

[37] Taherdoost H., Madanchian M. (2023). Multi-Criteria Decision Making (MCDM) Methods and Concepts. Encyclopedia.

[38] Si S.-L., You X.-Y., Liu H.-C., Zhang P. (2018). DEMATEL Technique: A Systematic Review of the State-of-the-Art Literature on Methodologies and Applications. Math. Probl. Eng..

[39] López-Nava I.H., Muñoz-Meléndez A. (2016). Wearable Inertial Sensors for Human Motion Analysis: A Review. IEEE Sens. J..

[40] McAdams E., Krupaviciute A., Gehin C., Grenier E., Massot B., Dittmar A., Rubel P., Fayn J. Wearable Sensor Systems: The Challenges. Proceedings of the 2011 Annual International Conference of the IEEE Engineering in Medicine and Biology Society.

[41] Spirescu M., Dumitru S., Constantinesu A., Badea C. (2017). Human–Robots Safe Cooperation in an Integrated Approach. Int. J. Mechatron. Appl. Mech..

[42] Drăgoi M.-V., Nisipeanu I., Frimu A., Tălîngă A.-M., Hadăr A., Dobrescu T.G., Suciu C.P., Manea A.R. (2024). Real-Time Home Automation System Using BCI Technology. Biomimetics.

[43] Constantin A., Badea C.R., Ancuta P.-N., Atanasescu A.I., Badea F., Badea S.I., Negrea C.S., Cioboată D.D. (2024). Research Related to an Optimized Design of a Simple Potentiometric Method for Real-Time Monitoring and Detecting the Human Physiological Posture. Proceedings of the International Conference on Reliable Systems Engineering (ICoRSE), Bucharest, Romania, 5–6 September 2024.

[44] Avramova T., Peneva T., Ivanov A. (2025). Overview of Existing Multi-Criteria Decision-Making (MCDM) Methods Used in Industrial Environments. Technologies.

[45] Hsieh Y.-F., Lee Y.-C., Lin S.-B. (2016). Rebuilding DEMATEL Threshold Value: An Example of a Food and Beverage Information System. SpringerPlus.

[46] Nechita R.-M., Deselnicu D.-C., Istriţeanu S.-E., Băjenaru V.-D. (2025). Analyzing Critical Factors for the Automotive Industry’s Transition to a Circular Economy: A Multi-Attribute Decision-Making Analysis Approach. Appl. Syst. Innov..

[47] Shieh J.-I., Wu H.-H., Huang K.-K. (2010). A DEMATEL Method in Identifying Key Success Factors of Hospital Service Quality. Knowl.-Based Syst..

[48] Wang F.-Y., Xu Y., Luo L.Y.-Y., Liang H.-B., Jiang Y.-P., Bai Z.-Q., Huang M.-Z., Wong A.Y.-L., Yang L., Zhang M. (2025). Can Wearable Real-Time Biofeedback Gait Training Devices Improve Gait Speed, Balance, Functional Mobility and Activities of Daily Living (ADL) in Individuals Post-Stroke? A Systematic Review and Meta-Analysis of Randomized Controlled Trials. J. Neuroeng. Rehabil..

[49] Alzahrani A., Aljohany M., Alsirhani H. (2026). Real-Time Wearable Biomechanics Framework for Sports Injury Prevention and Rehabilitation Optimization. Sci. Rep..

[50] Caña-Pino A., Holgado-López P. (2025). Wearable-Sensor and Virtual Reality-Based Interventions for Gait and Balance Rehabilitation in Stroke Survivors: A Systematic Review. Signals.

[51] Chen C.-T., Chu C.-C. (2024). A Fuzzy Method for Exploring Key Factors of Smart Healthcare to Long-Term Care Based on Z-Numbers. Mathematics.

[52] Esquivel K.M., Gillespie J., Kelly D., Condell J., Davies R., McHugh C., Duffy W., Nevala E., Alamäki A., Jalovaara J. (2023). Factors Influencing Continued Wearable Device Use in Older Adult Populations: Quantitative Study. JMIR Aging.

[53] Yuan W., Zhao H., Yang X., Han T., Chang D. (2024). Toward Dynamic Rehabilitation Management: A Novel Smart Product-Service System Development Approach Based on Fine-Tuned Large Vision Model and Fuzzy-Dematel. Adv. Eng. Inform..

[54] Cen C., Peng H., Li X., Zhang Z., Hu J., Wang Z., Jiang T. (2025). Interactive Interfaces and Wearable Technologies for Enhancing Health Management among Older Adults: A Systematic Review. Front. Med..

[55] Liao C.-W., Yao K.-C., Wang C.-H., Hsieh H.-H., Wang I.-C., Ho W.-S., Huang W.-L., Huang S.-H. (2025). Fuzzy Delphi and DEMATEL Approaches in Sustainable Wearable Technologies: Prioritizing User-Centric Design Indicators. Appl. Sci..

[56] Maček D., Magdalenić I., Begičević Ređep N. (2021). A Model for the Evaluation of Critical IT Systems Using Multicriteria Decision-Making with Elements for Risk Assessment. Mathematics.

